# Protein Assistants of Small Ribosomal Subunit Biogenesis in Bacteria

**DOI:** 10.3390/microorganisms10040747

**Published:** 2022-03-30

**Authors:** Elena Maksimova, Olesya Kravchenko, Alexey Korepanov, Elena Stolboushkina

**Affiliations:** Institute of Protein Research, Russian Academy of Sciences, 142290 Pushchino, Russia; maksimova.em@vega.protres.ru (E.M.); olesyak@vega.protres.ru (O.K.)

**Keywords:** ribosome biogenesis, 30S subunit maturation, 17S rRNA folding, assembly factors, RbfA, YjeQ (RsgA), Era, KsgA (RsmA), RimJ, RimM, RimP, Hfq, cryo-EM (cryo-electron microscopy)

## Abstract

Ribosome biogenesis is a fundamental and multistage process. The basic steps of ribosome assembly are the transcription, processing, folding, and modification of rRNA; the translation, folding, and modification of r-proteins; and consecutive binding of ribosomal proteins to rRNAs. Ribosome maturation is facilitated by biogenesis factors that include a broad spectrum of proteins: GTPases, RNA helicases, endonucleases, modification enzymes, molecular chaperones, etc. The ribosome assembly factors assist proper rRNA folding and protein–RNA interactions and may sense the checkpoints during the assembly to ensure correct order of this process. Inactivation of these factors is accompanied by severe growth phenotypes and accumulation of immature ribosomal subunits containing unprocessed rRNA, which reduces overall translation efficiency and causes translational errors. In this review, we focus on the structural and biochemical analysis of the 30S ribosomal subunit assembly factors RbfA, YjeQ (RsgA), Era, KsgA (RsmA), RimJ, RimM, RimP, and Hfq, which take part in the decoding-center folding.

## 1. Introduction

The ribosome is a large ribonucleoprotein complex responsible for the protein synthesis in all cells. The ribosome comprises small and large subunits containing several ribosomal RNAs (rRNAs) and dozens of proteins (r-proteins). Defects in the ribosome assembly lead to errors in translation and cause severe phenotypes up to cell death [1]. The essentiality of this process makes it a prospective target in the fight against pathogenic bacteria to improve antibacterial therapy. Faulty ribosome biogenesis underlies a spectrum of genetic diseases in humans and is called ribosomopathy [2]. Therefore, ribosome biogenesis is a fundamental process whose accuracy and efficiency is crucial.

**Figure 1:**
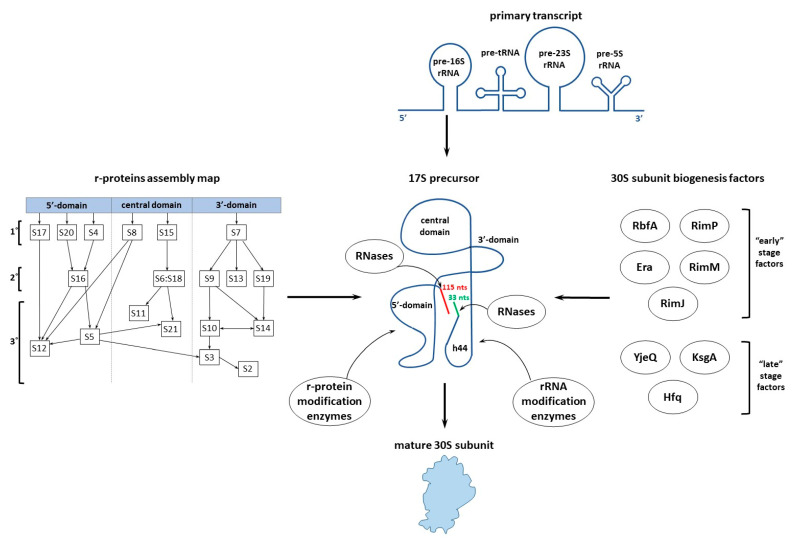
Main steps of the small ribosomal subunit (30S) assembly in bacteria. The steps are described in the main text in more detail. Hierarchical incorporation of the ribosomal proteins corresponds to Nomura assembly map [6]. Unprocessed 5′- and 3′-ends of the 17S rRNA are shown in red and green, respectively.

Ribosome biogenesis is complicated multistage process. The basic steps of ribosome assembly are: (1) the transcription, processing, folding, and modification of rRNA; (2) the translation, folding, and modification of r-proteins; and (3) consecutive binding of ribosomal proteins to rRNAs [3]. All these steps must occur in a certain order. The biogenesis of bacterial ribosomal subunits begins with the transcription of the primary rRNA transcript, which contains the precursors of 16S, 23S, and 5S rRNAs, and some tRNAs (Figure 1) [4,5]. Precursors of rRNAs and tRNAs fold cotranscriptionally prior to their excision from the primary transcript. Presence of unprocessed 5′- and 3′-ends in the rRNA precursors is critical to gain the native structure of ribosomal subunits in vivo. Therefore, the rRNA processing occurs at the final stage of the ribosome biogenesis. However, incorporation of r-proteins and modifications of the r-proteins and rRNAs by specific enzymes occur in concert with the rRNA folding (Figure 1). The r-proteins associate with rRNAs in a hierarchical manner and direct the folding of the rRNAs [6].

During the late assembly steps; for instance, the folding of functional centers of the ribosome, kinetic traps are abundant. The local minima in the folding landscape correspond to erroneous RNA conformations that appear due to degenerate local interactions [7]. At physiological conditions, these conformations can rearrange into the native structure, but rather slowly. Ribosome assembly factors are thought to avoid such kinetic traps, thus allowing the correct ribosome maturation.

In other words, ribosome assembly factors limit the folding pathways by assisting proper rRNA folding and protein–RNA interactions [3]. In addition, these factors may sense the checkpoints during the assembly. Ribosome biogenesis factors (also called ribosome assembly factors) are a broad spectrum of proteins that facilitate the formation of functional centers of the ribosome. Assembly factors include GTPases, RNA helicases, endonucleases, modification enzymes, molecular chaperones, etc. [1]. Some ribosome assembly factors have overlapping functions, and such redundancy is believed to be useful for the production of functional and mature ribosomes. Inactivation of these maturation factors leads to the accumulation of unprocessed rRNAs and, as a result, the production of defective ribosomes that cause translational errors and severe phenotypes [1].

**Figure 2:**
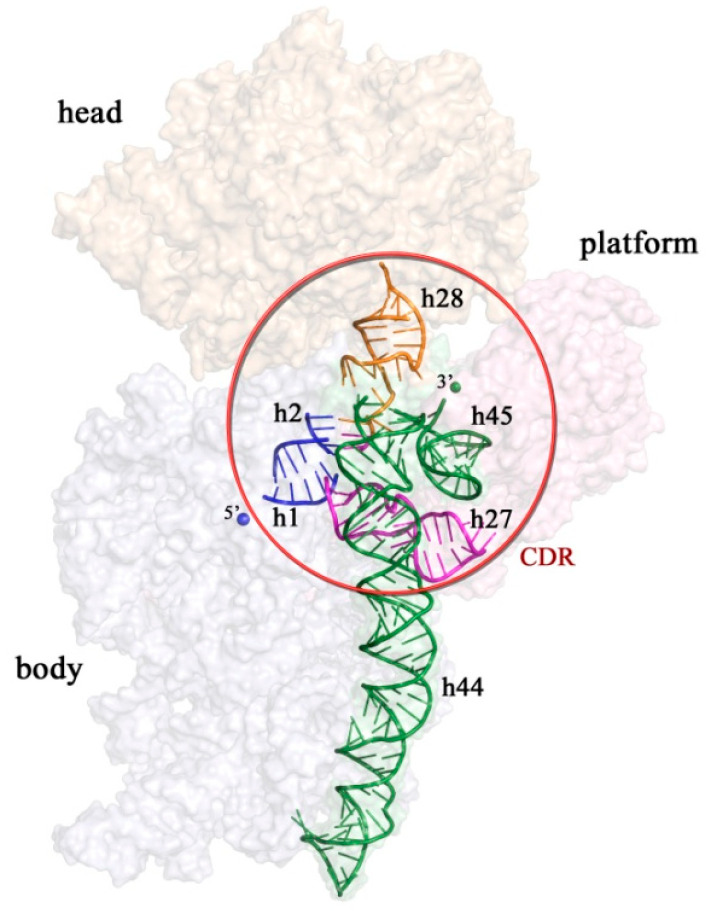
Surface of the 30S subunit of *Escherichia coli* (PDB ID: 7OE1) (inter-subunit interface view). Distinct structural domains of the 30S subunit (the head, the body, and the platform) are shown in light wheat, pale white, and light pink, respectively. The helices of the decoding center (CDR)—h1, h2, h27, h28, h44, and h45—are shown as illustrated models.

Here, we focused on the bacterial ribosome biogenesis factors that assist the production of the small ribosomal subunit (the 30S): RbfA, RimM, RimP, Hfq, Era and YjeQ (RsgA), the methyltransferase KsgA (RsmA), and the acetyltransferase RimJ. These assembly factors allow the functionally important region of the 30S ribosomal subunit—the decoding center (CDR)—to adopt the correct fold. CDR controls the correctness of the codon–anticodon base pairing [8]. The 30S subunit is composed of one rRNA molecule (16S rRNA) and about 21 r-proteins. It is organized into four distinct structural domains: the body (5′ domain), the platform (central domain), the head (3′ major domain), and helix 44 (h) with h45 (3′ minor domain) [9]. The decoding center is located between the body and the head of the 30S subunit. It includes the upper part of helix 44, helix 45, the switch helix 27, the neck helix 28, and the central pseudoknot (PK), consisting of helices 1 and 2 (Figure 2) [9]. The PK connects the head, the body, and the platform, and stabilizes the 30S subunit. The helix 44 is an essential segment of the 16S rRNA that is directly involved in the mRNA decoding and in the formation of two inter-subunit bridges (B2a and B3) that, among others, participate in association with the large ribosomal subunit (50S) [10,11].

There is a great body of data on the effects of the overproduction of an assembly factor on the defects caused by the lack of another one [12,13,14,15,16]. These, along with the cryo-EM analysis of immature 30S particles that accumulated in strains deficient in different assembly factors [14,16,17,18,19,20,21], allowed us to conditionally divide the factors into “early”-stage and “late”-stage ones. Both groups of the 30S assembly factors take part in the formation of the decoding center. “Early”-stage factors assist in formation of the central pseudoknot, including folding of the head, whereas “late”-stage factors facilitate positioning of the helix 44 at a later stage. RbfA, RimJ, RimM, RimP, and Era belong to the group of “early”-stage factors. YjeQ, KsgA and Hfq are “late”-stage factors (Figure 1). Moreover, several “early”-stage factors assist the 30S assembly throughout the entire process. In this review, we present the structural and biochemical analysis of the selected assembly factors.

## 2. 30S Ribosomal Subunit Biogenesis Factors

### 2.1. RbfA

**Figure 3:**
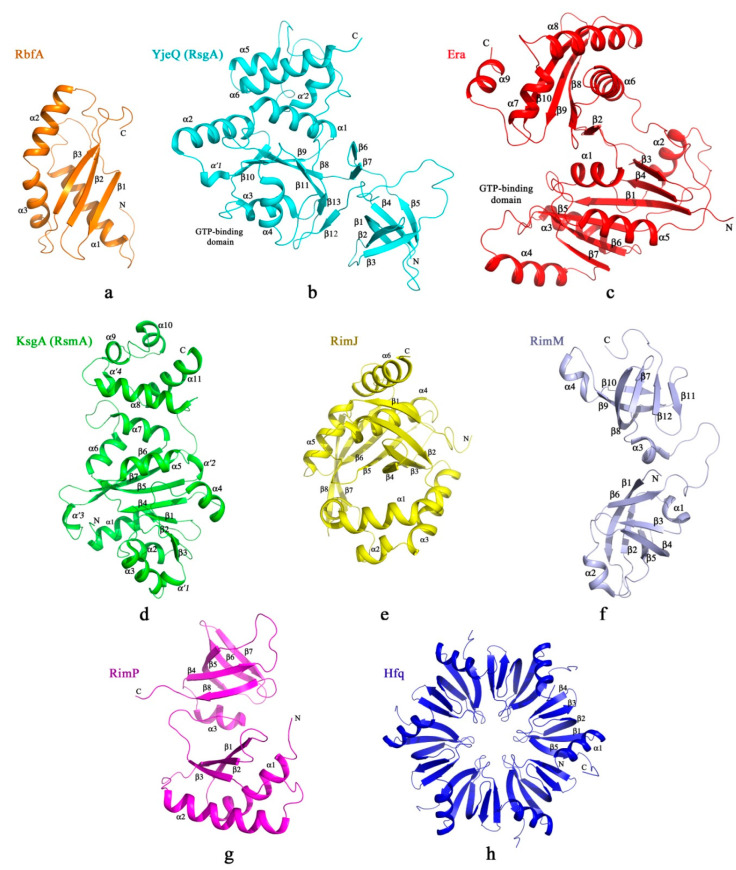
Domain organization of the 30S assembly factors. Secondary structure elements, α-helices (α), β-strands (β), and 3_10_ helices (α’) are numbered according to their relative position in the polypeptide sequence. Crystal structures of: (**a**) RbfA (PDB ID: 1KKG); (**b**) YjeQ (RsgA) (PDB ID: 1U0L); (**c**) Era (PDB ID: 1EGA); (**d**) KsgA (RsmA) (PDB ID: 1QYR); (**e**) RimJ (PDB ID: 6C30); (**f**) RimM (PDB ID: 2DYI); (**g**) RimP (PDB ID: 7NAT); (**h**) Hfq (PDB ID: 1HK9).

**R**ibosome-**b**inding **f**actor **A** (RbfA) is a small single-domain protein (13–15 kDa) consisting of three α helices and three β strands with a type-II K homology (KH)-domain fold topology (Figure 3a) [22]. The KH domain has a characteristic helix–turn–helix consensus GxxG motif involved in the RNA recognition [23,24]. Following the GxxG binding motif of the KH domain, RbfA exhibits a preference for the AxG sequence [22]. RbfA is found in the majority of bacteria and archaea, and RbfA orthologs occur in eukaryotic mitochondria and chloroplasts [25,26]. Deletion of the *rbfA* gene results in slow-growing bacteria and the accumulation of free ribosomal subunits and unprocessed 16S rRNA (17S rRNA) [12,27]. RbfA was initially found as a multicopy suppressor of the cold sensitivity C23U mutation of 16S rRNA, locating at the 5′ terminal helix (h1) [28]. RbfA overcomes the translational block at low temperature, promoting the correct folding and maturation of the 5′-end of the 16S rRNA [29].

**Figure 4:**
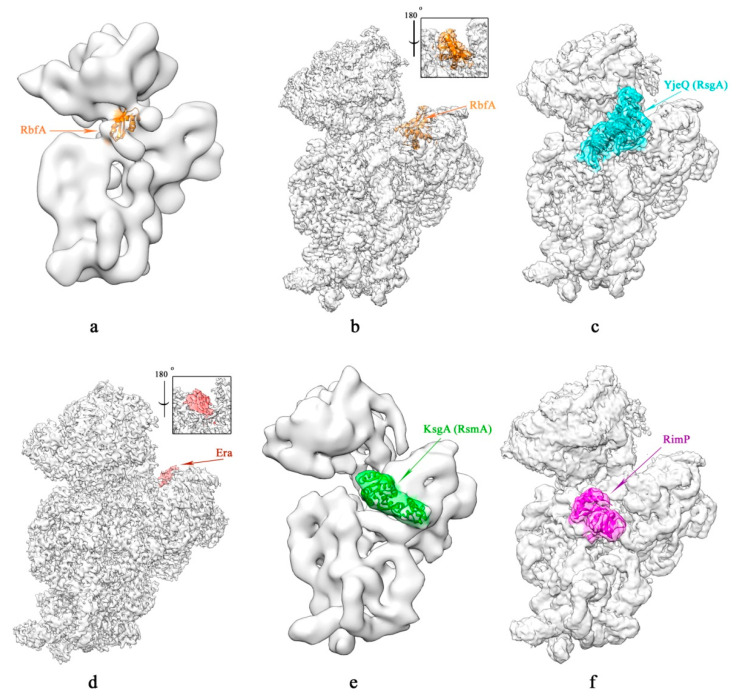
Cryo-EM structures of 30S subunits in complex with different ribosome biogenesis factors: (**a**,**b**) 30S•RbfA (EMDB ID: 1413; PDB ID: 2R1C and EMDB ID: 12243; and PDB ID: 7BOH, respectively); (**c**) 30S•YjeQ (RsgA) (EMDB ID: 12245; PDB ID: 7NAR); (**d**) 30S•Era (EMDB ID: 0484); (**e**) 30S•KsgA (RsmA) (EMDB ID: 2017; PDB ID: 4ADV); (**f**) 30S•RimP (EMDB ID: 12247; PDB ID: 7NAT).

Two cryo-EM structures of the 30S subunit in complex with RbfA did not reveal any direct interactions between RbfA and helix 1 of 16S rRNA [29,30]. Moreover, these structures showed different RbfA localization on the small ribosomal subunit (Figure 4a,b). RbfA binds in the neck region (helix 28), being buried within the cleft between the head and the body of the 30S subunit [29,30]. In one structure, RbfA is localized at the interface of the 30S subunit: the C-terminus of RbfA extends toward the 5′-end of the 16S rRNA, and the N-terminal RNA-binding interhelical kink with an AxG motif (helix α2-turn-helix α3) faces the junction between h44 and h45, in that the top of helix 44 is displaced [29]. In another structure, the RNA-binding AxG motif of RbfA interacts with the 3′-end of the 16S rRNA [30]. Interestingly, for RbfA binding, the 3′-end of the 16S rRNA must be in the exit channel. The rearrangement of the 3′-end of the 16S rRNA from the entry to the exit of the mRNA channel is accompanied by reorganization of the helix 28 [9]. It has been suggested that RbfA promotes the conversion of h28 to a mature state, and holds the 3′-end of the 16S rRNA at the exit of the mRNA channel [30]. The upper part of h28 is displaced, and the lower end of h28 is destabilized, thus keeping h28 from adopting a fully matured conformation [30]. Nucleotides of the disordered lower part of the helix 28 play an important role in stabilizing the h44/45 linker in the mature 30S subunit. Therefore, RbfA delay the folding of the h44/45 linker and promote the correct folding of adjacent h1 via h28. It was shown that RbfA acts as a “gatekeeper” to prevent premature entry of pre-30S subunits into the translation cycle. RbfA inhibits protein synthesis by pre-30S subunits in the presence of translation initiation factor IF3 [31]. IF3 was able to release RbfA from mature 30S subunits, but not from immature 30S particles. These findings indicate that RbfA may act as a “gatekeeper” if RbfA interacts with the 3′-end of the 16S rRNA in the mRNA channel exit. Therefore, localization of RbfA on the 30S subunit in the second structure is preferable. The cryo-EM analysis of Δ*rbfA* pre-30S subunits (immature 30S particles accumulated in an *rbfA* null strain) revealed a number of assembly intermediates: from the particles with a completely unresolved head domain and unfolded central pseudoknot to almost mature 30S subunits with the well-resolved body, platform, and head domains, and a partially distorted helix 44 [17]. These data were in agreement with the results of X-ray-induced hydroxyl radical footprinting of the 30S assembly intermediates that accumulate in the Δ*rbfA* cells [32], as well as with a cryo-EM reconstruction of the 30S intermediates from a Δ*rbfA*Δ*rsgA* double mutant [18], which revealed the excessive flexibility of the 3′ domain, including h44 and h45. In addition, it was shown that the formation of the central pseudoknot might promote stabilization of the head domain, likely through the RbfA-dependent maturation of the neck helix 28 [17]. The presence of two predominant classes of 30S intermediates accumulated in the Δ*rbfA* cells indicated that RbfA is more deeply involved in the maturation process and can act at two distinctive 30S assembly stages: early formation of the central pseudoknot, including folding of the head; and positioning of the helix 44 in the decoding center at a later stage.

### 2.2. YjeQ (RsgA)

YjeQ is a ribosome-dependent GTPase (also known RsgA–**R**ibosomal **s**mall subunit **G**TPase **A**), the activity of which is stimulated by the mature 30S subunit [33,34,35] and inhibited by the stringent response mediators pppGpp and ppGpp [27,28]. The ribosome assembly factor YjeQ is found in Gram-negative bacteria, and its homolog YloQ is present in Gram-positive bacteria. There are no YjeQ/YloQ counterparts in eukaryotes and in archaea. The *yjeQ* gene is important for bacterial growth [36]. The bacterial cells with a knockout of the *yjeQ* or *yloQ* gene are viable but grow very slowly and accumulate the 17S rRNA [19,37,38].

YjeQ (about 33 kDa in *Thermotoga maritima*) consists of three domains (Figure 3b) [39,40,41,42]. The N-terminal or OB-fold domain (oligonucleotide/oligosaccharide-binding) represents a β-barrel. N-terminal amino acid residues from 1 to 20 are essential for tight binding to the 30S ribosomal subunit [33]. The central GTPase domain is a Rossmann fold with a unique circular permutation: instead of the standard order G1–G2–G3–G4–G5, which is characteristic of TRAFAC (**TRA**nslation **FAC**tor) GTPases, it possesses a G4–G5–G1–G2–G3 motif because the N-terminal and C-terminal loops are swapped [39,40,41,42]. As a result of this rearrangement, the C-terminal domain may have a direct mechanical effect on the GTP hydrolysis in YjeQ. The C-terminal part consists of one 3_10_-helix and the two α-helices with the zinc-finger motif Cx(F/Y)xxCxHxx(E/D)xxC. [39,40,41,42]. These cysteines and histidine are located in the loop connecting two α-helices and coordinate a zinc ion. The zinc finger contributes to the structural stability of the C-terminal domain.

Cryo-EM analysis of immature 30S particles from the Δ*yjeQ* cells showed that late assembly r-proteins S2 and S21 were underrepresented [18,19,20]. Helix 44 of the 16S rRNA was distorted, and the extent of the distortion varied from class to class of the Δ*yjeQ* 30S assembly intermediates. The class with the largest h44 distortion showed outward displacement of the upper part of helix 44 [19]. On the other hand, the class with fewer h44 distortions was correlated with the increase in S2 density amount in the cryo-EM maps, indicating that the decoding center was closer to the one found in mature 30S subunits. Most likely, YjeQ is involved in the positioning of the helix 44 within the decoding center at a late stage of the 30S assembly.

As for the YjeQ binding site on the ribosome, there are currently five cryo-EM structures of the mature 30S subunit with YjeQ [30,43,44,45,46]. The first two structures placed YjeQ at the same site on the 30S subunit, but in the opposite orientations [43,44]. All subsequent 30S subunit•YjeQ structures confirmed only one of the two orientations of YjeQ (Figure 4c) [30,45,46]. The C-terminal domain of YjeQ forms a bridge between the head (h29 and h31) and the platform of the 30S subunit. The N-terminal OB-fold part of YjeQ interacts with h18 and h44 of the decoding center, while the GTPase domain contacts h44 and h24 of the platform. Both GTPase and OB-fold domains of YieQ resemble a clamp around the top of helix 44, limiting its mobility. A1492, along with the other universally conserved A1493, monitors the geometry of the codon–anticodon base pairing in the decoding center, and bulges out from the helix 44. This conformation of A1492 is similar to that adopted by A1492 and A1493 during proofreading of the codon–anticodon pairing [47]. The OB-fold domain of YjeQ stabilizes the A1492 position via hydrophobic and π-stacking interactions [30,45,46]. It should be noted that the 70S ribosome dissociates in the presence of YjeQ [37]. These data suggested that YjeQ sterically prevents premature 30S subunit from association with the 50S subunit and may act as a checkpoint protein that tests maturing 30S subunits for their ability to proofread. After correct 30S subunit maturation, the factor YjeQ dissociates, and mature 30S subunits become available for the translation. The YjeQ release from 30S subunit is accompanied by GTP hydrolysis [48]. Superimposition of YjeQ•GMP-PNP (nonhydrolyzed GTP analog) and YjeQ•GDP structures revealed the reorientation of both the N-terminal OB-fold and C-terminal zinc-finger domains [40,45]. The trigger for GTP hydrolysis is conformational rearrangements of the helix 44 top that interacts with switches of the GTPase domain of YjeQ.

A study on the compensation of slow growth of the Δ*yjeQ* strain revealed suppressor mutations in the *rbfA* gene [13]. It turned out that these mutations promoted spontaneous dissociation of RbfA from the 30S subunit, and only RbfA mutants, but not the wild-type RbfA, suppressed the 30S subunit maturation defects in the Δ*yjeQ* cells. Prolonged association of RbfA with the 30S subunit aggravates defects in the *yjeQ* null mutant. According to the structures of the 30S subunit•RbfA complex [29,30], it was suggested that proteins RbfA and YjeQ had overlapping sites. It was shown that the GTP-bound state of YjeQ enhanced the release of RbfA from the mature 30S subunit [13], and the C-terminal α-helix of YjeQ was essential in this process [35]. However, IF3 may be the dominant factor that releases RbfA during stationary phase [32], when the GTPase activity of YjeQ is inhibited by the alarmone (p)ppGpp [49].

### 2.3. Era

The ribosome biogenesis factor Era belongs to the Ras-like small GTPase family (*E. coli* **Ra**s-like) [50]. Ras is an acronym for rat sarcoma, in which the first members of the Ras family were discovered [51]. Era is a vital GTP-binding protein found in almost all bacteria [52,53,54,55,56]. Era is required for growth and cell division in bacteria [52,57,58]. Era homologs were found in *Drosophila*, mice, *Homo sapiens* [58], the plant *Antirrhinum majus* [59], the roundworm *Caenorhabditis elegans* [58], and in certain archaea [60]. Era depletion in eukaryotic cells led to G1 phase cell-cycle arrest and apoptosis [61]. In bacteria, Era deficiency results in increased levels of free 30S and 50S ribosomal subunits [62], accumulation of unprocessed 16S rRNA, and inhibition of translation [63]. The Era overexpression compensates for the *rbfA* deletion that indicates Era participation in the maturation of the 30S subunit [27]. Era function probably overlaps RbfA.

Era is a small two-domain protein (34 kDa in *E. coli*) (Figure 3c). The N-terminal GTP-binding domain of Era represents a six-stranded β-sheet surrounded by five α-helices [64,65]. It was shown that serine and threonine residues in the _33_ISITSR_38_ sequence of the β2 strand of *E. coli* Era could be phosphorylated [66]. The regulation of the Era activity via autophosphorylation is vital. Double substitution of Thr36Ala and Ser37Ala [66] and a point mutation of Ser34Pro in *E. coli* Era [67] were lethal. The C-terminal domain of Era consists of a three-stranded β-sheet and three α-helices with αββααβ topology, which is characteristic of the KH type II domain fold implicated in RNA binding [23]. Mutations in the GlyxxGly consensus of the KH domain of Era inhibited the Era’s binding to the 16S rRNA [68]. The two domains of Era are connected by a flexible linker, the length of which is important for ribosome biogenesis [63]. An insertion mutation with eight amino acid residues (AAANAAAN) inserted into the Era’s linker led to loss of suppression of the 30S maturation defects in the Δ*rbfA* strain. 

The cryo-EM structure of the mature 30S subunit in complex with Era demonstrated that the Era factor was located in the cleft between the head and platform, the opposite face of helix 28 from RbfA (Figure 4d) [69]. Era bound the anti-Shine–Dalgarno (anti-SD) _1531_AUCACCUCCUUA_1542_ sequence at the 3′-end of the 16S rRNA. The _1531_AUCA_1534_ region “wraps around” side chains of two lysine residues of the consensus _250_GlyLysLysGly_253_ loop in the KH domain of Era [64]. Moreover, the cryo-EM structure of 30S subunit•Era complex revealed that binding of Era and ribosomal protein S1 was mutually exclusive [69]. In addition, Era may associate with the RNase YbeY and the RNA-helicase CshA [70]. Apparently, one of the roles of Era is to protect the anti-SD sequence from cleavage during the processing of the 3′-end of the 17S rRNA, and therefore block the duplex formation between the anti-SD of the 17S rRNA and the SD sequence of an mRNA, preventing premature translation. 

Cryo-EM analysis of immature 30S subunits obtained from Era-depleted *E. coli* cells revealed that helices 23 and 24 of the platform failed to adopt the mature conformation [14]. In addition, helices 44 and 45 were disordered in the Δ*era* 30S particles, indicating that their maturation requires proper stabilization of helices 23 and 24. Interestingly, the 70S ribosome treatment with Era promoted ribosome dissociation and caused destabilization in h44 of the decoding center of the 30S subunit. The Era-induced destabilization of h44 inhibited YjeQ binding to the 30S subunit. Conversion of helix 44 to an immature state indicated that Era plays a role in ribosome homeostasis, including the reactivation of hibernating ribosomes or triggering of the ribosome degradation [14]. Still, it does not explain how Era suppress defects of the 30S maturation in Δ*yjeQ* cells [71]. In addition, it was shown that, similarly to YjeQ, Era is a target for (p)ppGpp-mediated stress response, and its GTPase activity is inhibited by the (p)ppGpp alarmones [49].

Numerous experiments were devoted to studying the GTPase activity of Era, but the molecular mechanisms of Era’s functional cycle are still unclear. The kinetics of guanine nucleotide binding and exchange, and GTP hydrolysis in purified Era, have been measured. Era demonstrated an extremely rapid guanine nucleotide exchange rate, but the GTP hydrolysis occurred with minutes in vitro [72]. The GTP-hydrolyzing activity of Era was stimulated 3- to 12-fold in the presence of the anti-SD _1531_AUCACCUCCUA_1542_ fragment of 16S rRNA (different regions of this anti-SD sequence impact the GTP-hydrolyzing activity differently) [65]. Comparison of crystal structures of four Era functional states (ternary complex Era•GDPNP•5′-AUCACCUCCUUA-3′, binary complexes Era•GDPNP, Era•GDP, and apo-Era) revealed only two conformations of the Era protein [64,65]. Era•GDPNP•5′-AUCACCUCCUUA-3′ and Era•GDPNP shared conformation where the GTP-binding site was closed, and the RNA-binding site was exposed, indicating that the RNA binding did not cause any considerable conformational changes in the protein [64]. Similarly, Era•GDP and apo-Era adopted the same conformation, but this time the GTP-binding site was opened, and the RNA-binding site of the KH domain was closed. This suggested that the GDP release did not result in conformational changes, while two different conformations of Era indicated that GTP binding and GTP hydrolysis caused dramatic conformational changes in the protein. Interestingly, none of these conformations were observed in the structure of the 30S-bound Era [69], so studies need to be continued.

### 2.4. KsgA (RsmA)

KsgA (also known as RsmA—**R**ibosomal RNA **s**mall subunit **m**ethyltransferase **A**) was identified upon selecting *E. coli* strains resistant to the aminoglycoside antibiotic kasugamycin due to the lack of A1518 and A1519 dimethylation of the 16S rRNA [73,74]. KsgA is (adenine-N6, N6-)-dimethyltransferase, which dimethylates the two adjacent adenosines of the helix 45, A1518 and A1519 [73]. Lack of A1518 and A1519 dimethylation caused severe damage to the translation [75,76]. A1518 and A1519 are universally conserved residues, and KsgA occurs in all three domains of life [77,78], suggesting that the modification of the two adjacent adenosine residues has an important role in cells. Nevertheless, *ksgA* was not required for the survival of *E. coli* [79], whereas its knockout was lethal in yeasts [80].

It was shown that deletion of *ksgA* substantially slowed cell growth of *E. coli* [81]. This effect was even more substantial at lower temperatures. The Δ*ksgA* cells accumulated free ribosomal subunits and 17S rRNA. Surprisingly, the overexpression of a methyltransferase-inactive form of KsgA (Glu66Ala) in the Δ*kgsA* strain led to a dramatically reduced growth rate at all temperatures, as well as accumulation up to 95% of free 30S subunits containing the 17S rRNA [81]. It turned out that catalytically inactive KsgA strongly associated with the pre-30S subunits and stalled the 17S rRNA processing, excluding premature subunits from the translation cycle. These data indicated that KsgA’s role goes beyond the methylation of the 16S rRNA, and KsgA is integrated into the network of other 30S assembly factors. So, methylation is a second function for KsgA, while its primary and critical role is monitoring the 30S subunit biogenesis. It was shown that KsgA had a low affinity for methylated 30S subunits [74]. The methylation was assumed to be a trigger for release of KsgA from assembling 30S subunits, and the release of KsgA was accompanied by conformational rearrangements that allowed final maturation and entrance into the translation cycle.

KsgA consists of two domains with 30 kDa molecular mass (for *E. coli*) (Figure 3d). The N-terminal catalytical domain of KsgA has a Rossmann-like fold and three additional 3_10_-helices [78]. This Rossmann-like fold consists of a central seven-stranded β-sheet sandwiched between three α-helices on one side and four on the other. The C-terminal domain of KsgA comprises four α-helices and one 3_10_-helix. It was shown that KsgA was unable to methylate 30S subunits in the translationally active conformation. At the same time, it could modify 30S subunits when they were in an experimentally well-established, translationally inactive conformation, indicating that KsgA recognized a specific conformational state of the 30S ribosomal subunit [82]. It was found to require the presence of a minimal set of ribosomal proteins for methylation: proteins of platform S6, S11, S15, S18; and body S4, S8, S16, S17 [82,83].

**Figure 5:**
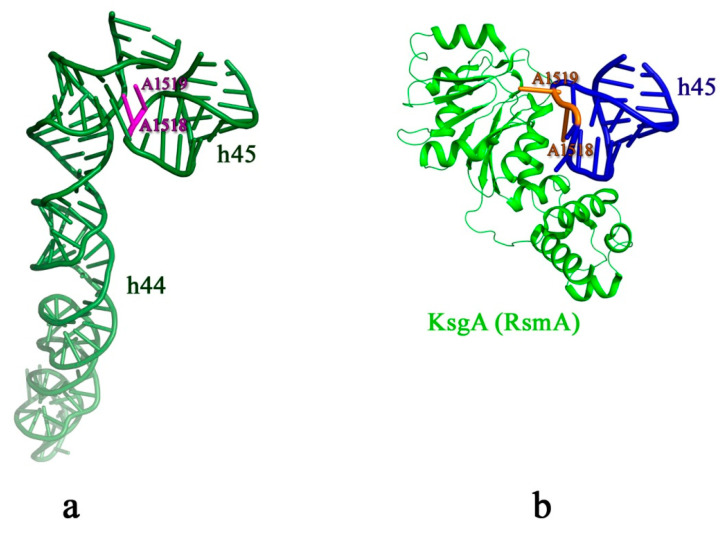
A1518 and A1519 dimethylation is described in the main text. (**a**) Illustration of h44 and h45 (forest green) of the *E. coli* 16S rRNA (PDB ID7OE1). A1518 and A1519 nucleotides are shown in magenta. (**b**) Illustration of h45 (blue) and KsgA (green) extracted from the published structure of the ΔkgsA pre-30S subunit•KsgA complex (PDB ID 7O5H). A1518 and A1519 are shown in orange.

The structures of the 30S ribosomal subunit with KsgA have been determined for mature 30S subunit incubated with KsgA after depletion of magnesium ions [30,84], and for the immature 30S subunit from the Δ*kgsA* cells also under low Mg^2+^ concentration conditions [85]. The mature 30S subunits could hold KsgA, as they mimicked the methylation-ready conformation under the low-magnesium conditions [84]. Cryo-EM data for the 30S subunit•KsgA complex were consistent with the results of hydroxyl-radical footprinting, according to which KsgA bound to the 30S subunit at the inter-subunit interface around the decoding center and the platform (Figure 4e) [86]. The C-terminal domain of KsgA bound to helices 27 and 24 of the 16S rRNA (platform), and the N-terminal domain of KsgA contacted the tetraloop of the helix 45 where A1518 and A1519 were located [30,84,85]. KsgA contacted the h45 so that the h44 could not access its native position [16,81]. The structure of the Δ*kgsA* 30S particles•KsgA complex revealed that A1518 and A1519 dimethylation of 16S rRNA could occur one after the other, without KsgA dissociation [85]. A1519 is positioned in the catalytic site of KsgA in an outward-flipped conformation, which allows it to be methylated (Figure 5) [85]. Apparently, A1518 also flips outward from the tetraloop into the catalytic site, replacing methylated A1519 from it. Likely, A1518 and A1519 dimethylation induces the packing interaction between helices 45 and 44 in the decoding center and is another checkpoint for the 30S assembly [87]. It was shown that the Δ*kgsA* strain overexpressing RbfA accumulated 70S-like particles with a reduced association ability and an impaired translational capacity [15]. In addition, the result of RbfA overexpression in the Δ*kgsA* strain was a dramatic loss of viability, even at standard growth temperatures. Due to the steric clash between KsgA and h69 of the 23S rRNA in the 50S subunit and the overlapping location with IF3, KsgA could prevent the 70S ribosome formation and initiation of translation until the methylation was accomplished [81].

### 2.5. RimJ

A number of ribosomal proteins in *E. coli* undergo post-translational modifications, but the functional significance of these modifications has remained unclear. Several enzymes perform post-translational modifications of ribosomal proteins. For example, enzymes RimJ, RimI, and RimL acetylated the N-terminal residues of ribosomal proteins S5, S8, and L7/L12, respectively, and had structural similarities [88,89]. To date, it has been shown that RimJ is associated with the ribosome biogenesis [90]. RimJ is a single-domain protein (23 kDa in *Mycobacterium smegmatis*) consisting of six α helices and eight β strands with a NAT-domain fold typical for **N**-**a**cetyl**t**ransferases (Figure 3e).

The protein S5 takes part in the formation of the functionally important structure of 30S subunit, the central pseudoknot of the decoding center [9]. It was shown that the point mutation Gly28Asp in the ribosomal protein S5 of *E. coli* caused a cold-sensitive phenotype: growth was not detectable for this mutant strain at a reduced temperature (20 °C), which could have indicated defects in the ribosome biogenesis [90,91]. Indeed, the S5 (Gly28Asp) mutant demonstrated increased amounts of free 30S and 50S subunits, accumulation of 17S rRNA, and reduced translation fidelity [90,91]. Deletion of *rimJ* exacerbated the growth defects of the S5 (Gly28Asp) mutant. Still, overexpression of RimJ suppressed cold sensitivity and partially alleviated anomalous ribosome profiles and mRNA misreading associated with the S5 (Gly28Asp) mutation [90]. Mass spectrometry analysis revealed that the S5 protein in both the wild-type and S5 (Gly28Asp) 30S subunits was acetylated even without RimJ overexpression [90]. Furthermore, the elimination of acetyltransferase function by site-directed mutagenesis of two conserved cysteine residues at positions 54 and 105 in *E. coli* RimJ did not disrupt the suppression activity of RimJ [90]. This RimJ mutant could still suppress the cold-sensitive phenotype of the S5 (Gly28Asp) strain. Hence, this indicated that the acetyltransferase activity is not required for the ability of RimJ to suppress the cold sensitivity caused by the Gly28Asp change in S5. Moreover, deletion of the *rimJ* gene slowed growth, decreased the amounts of total protein per cell, and caused defects in ribosomal function [92]. So, Rim may have an additional function as a ribosome assembly factor, taking part in the remodeling of the structure of the central pseudoknot for stable association of protein S5 with its subsequent acetylation.

It has been shown that RimJ did not interact with mature 30S and 50S subunits, nor did it interact with 70S ribosomes [90]. Moreover, RimJ did not acetylate free protein S5, and only modified S5 in the context of the pre-30S subunit [32,93]. It was found that the N-terminal acetylation of S5 occurred after the correct folding of the central pseudoknot of the pre-30S subunit [32,94]. This suggested that this modification flagged the formation of specific RNA–protein contacts during assembly. 

### 2.6. RimM

**Ri**bosome **m**aturation factor M (RimM) is conserved in bacteria. Rim-like proteins are present in eukaryotes such as *Arabidopsis thaliana*, *Anopheles gambiae*, *Plasmodium falciparum,* and *Plasmodium yoelii*, but were not found in archaea [95]. RimM is a small two-domain protein with a molecular weight of 21 kDa in *E. coli*. Each domain folds into a β-barrel structure (Figure 3f) (PDB ID: 2F1L; 2QGG; 2DYI; 3H9N; 3A1P); [96]. The N-terminal part of the protein RimM demonstrated extensive similarity to the KH-domain and contains two copies of the highly conserved GxxG motif involved in interactions with RNA [23]. Due to the structural similarity of the C-terminal domain of RimM to one of the subunits of the photosynthetic reaction center in purple bacteria, it has been called the PRC-barrel domain [97]. This domain is essential for the interaction with the r-protein S19 [95].

Bylund and coauthors first observed the ribosome-related function of RimM. They showed that *rimM* deletion led to slow cell growth and a reduced translational efficiency [98]. Later, they demonstrated that most parts of the 16S rRNA were not processed in cells with *rimM* deleted [12]. In addition, the *rimM* deletion aggravated the growth defect of the Δ*yjeQ* strain [71]. The genetic interaction between *rimM* and *yjeQ* was consistent with the hypothesis that RimM plays a role in 30S biogenesis and is related to YjeQ’s function. At the same time, overexpression of Era is not able to suppress a *rimM* disruption phenotype.

Suppressor mutations in Δ*rimM* and RimM-YY→AA strains were analyzed to study RimM’s involvement in 30S subunit maturation [12,95,99]. The RimM-YY→AA mutation in the PRC-barrel domain demonstrated reduced binding of RimM to the 30S subunit and accumulation of the 17S rRNA [95,99]. Suppressor mutations in Δ*rimM* and RimM-YY→AA strains eliminated the deficiency of 16S rRNA processing [12,95,99]. In addition, the Δ*rimM* knockout could be compensated by an increased gene dosage of *rbfA* [11]. Suppressor mutations in the Δ*rimM* strain seemed to affect the transcription or stability of the *rbfA* mRNA. In the RimM-YY→AA strain, suppressor mutations were detected in the *rimM* mRNA that increased RimM synthesis [99]. Furthermore, in both the Δ*rimM* and RimM-YY→AA strains, suppressor mutations were found in helices 31 and 33b of 16S rRNA, and in genes encoding r-proteins S13 and S19 [95]. These findings suggested that RimM is important for the maturation of the head region of the 30S subunit, which includes r-proteins S13 and S19 and helices 31 and 33b.

Direct evidence of RimM participation for the correct structure/conformation of the head domain of the 30S subunit was obtained using in vivo X-ray footprinting and cryo-electron microscopy [20,21,32]. Using a synchrotron X-ray beam to generate hydroxyl radicals in the cytoplasm revealed that nucleotides of helices 31 and 33 were exposed in the Δ*rimM* strain [32]. Cryo-EM analysis of Δ*rimM* pre-30S subunits showed a high rotation of the 3′-head domain relative to the body domain; under-representation of head r-proteins S10, S14, S13, and S19; and fragmented densities for helices 44 and 45 of the 16S rRNA [20,21]. Incubation of Δ*rimM* pre-30S subunits with RimM significantly reduced the flexibility of the head domain. However, helix 44 still displayed a large variability, indicating that RimM probably did not affect the final positioning of the helix 44 in the decoding center [21]. Furthermore, the kinetic data showed that RimM accelerated binding of some head domain r-proteins, S9 and S19, but inhibited interaction with the central pseudoknot-binding protein S12 [100]. Together, these results suggest that RimM does not simply recruit S19 to the complex. The RimM interaction with S19 results in the stabilization of the rRNA tertiary structure, holding the 3′-domain of the 16S rRNA in an open conformation that allows the correct folding of the helices in the head domain of the 30S subunit. Most likely, RimM reduces the misfolding of the 3′-domain of the 16S rRNA.

### 2.7. RimP

In *E. coli,* the *rimP* gene encoding the RimP protein is located in the same operon as *rbfA* [16]. In Gram-negative bacteria, *rimP* knockout causes slow cell growth [16,101]; whereas in Gram-positive *Streptococcus pneumoniae*, the deletion of *rimP* is lethal [102], and in *Mycobacterium fortuitum*, RimP is required for survival under stress conditions [103]. RimP has not been identified in eukaryotes and archaea, but it is highly conserved in bacteria. RimP is a small globular protein (approximately 17 kDa in *E. coli*) that consists of two domains; each domain has an α/β-fold (Figure 3g) [102,104,105]. The N-terminal domain contains two α-helices and a three-stranded β-sheet, whereas the C-terminal part consists of a single α-helix and a five-stranded β-sheet. The N-terminal domain of RimP is structurally similar to RbfA, and the C-terminal domain has an Sm fold that is characteristic of proteins belonging to Sm/Lsm superfamily The representative of this superfamily in bacteria is the Hfq protein (whose role in the ribosome biogenesis is addressed in the next section). Unlike the Hfq protein, which forms a hexameric ring, RimP exists in a monomeric form.

Deleting *rimP* in *E. coli* caused a temperature-sensitive growth phenotype, which differentiated RimP from other 30S subunit biogenesis factors [16]. In addition, at higher temperatures, the Δ*rimP* strain exhibited the typical ribosome biogenesis defects: a reduced amount of 70S ribosomes, an increase in the amount of free 30S and 50S ribosomal subunits, and accumulation of immature 16S rRNA. Interestingly, none of the known 30S maturation factors (RimM, RbfA, Era, KsgA, and YjeQ) suppressed the defects of the Δ*rimP* strain [16]. Moreover, the N-terminal domain of RimP (similarly to RbfA) did not suppress the slow growth of a *rbfA* null mutant [16]. This was probably due to different surface charges of the proteins, which were predominantly negative for the N-terminal domain RimP and positive for RbfA. Therefore, the RimP protein may act independently of the other assembly factors and is crucial at higher temperatures. Similarly, to Hfq, a robust Sm fold apparently preserves the native structure of RimP at high temperatures. It may be assumed that RimP is overexpressed under heat-shock conditions to maintain efficient ribosome biogenesis at higher temperatures.

Cryo-EM analysis of pre-30S subunits accumulating in the Δ*rimP* strain showed a wide range of 30S assembly intermediates from “early” particles with an almost missing cryo-EM density for the head to nearly mature 30S subunits with body, platform, and head domains and a distorted helix 44 [106]. The presence of intermediates with an unformed central pseudoknot and depletion of S5 and S12 proteins interacting with helices of central PK is a characteristic feature of Δ*rimP* pre-30S particles. Quantitative MS studies on *E. coli* revealed that RimP accelerated the binding of the S5 and S12 to the 16S rRNA in vitro [100]. It was suggested that RimP takes part in stabilization of the central PK at the early stages of the 30S subunit maturation, and this process may occur before the head domain assembly [106]. Cryo-EM analysis of complexes of RimP with mature 30S subunits simulating different assembly stages (inactive/active forms and in combination with different 30S assembly factors) revealed that RimP was bound to the t majority of the 30S assembly states (Figure 4f) [30]. This indicated that RimP (similarly to RbfA) is deeply involved in the 30S subunit maturation process and can assist in the formation of the central pseudoknot and later stages of the 30S assembly. RimP interacts with the helix 44 in the vicinity of KsgA-binding site on the 30S subunit, and apparently prevents A1492 and A1493 residues at the top of h44 from approaching S12, which maintains translation fidelity [30]. So, RimP, together with KsgA, delay h44 positioning at the 30S subunit interface at the late stage of the 30S assembly.

### 2.8. Hfq

The host factor for Qβ replication (Hfq) is a new ribosome biogenesis factor. Hfq was discovered to be one of the *E. coli* proteins required for in vitro replication of the Qβ bacteriophage [107]. Hfq melts the secondary structure of the phage RNA, facilitating its interaction with the Qβ replicase [108]. Hfq is a highly conserved thermostable hexameric protein with a 12 kDa molecular mass for the monomer in *E. coli*. [109]. The Hfq monomer possesses a characteristic Sm fold that consists of the N-terminal α-helix and the five-strand antiparallel β-sheet (Figure 3h) [110]. It belongs to the Sm/Lsm superfamily of eukaryotic and archaeal proteins [111,112,113]. In eukaryotes, these proteins form ringlike hexa/heptamers, and are components of the spliceosome in which they mediate RNA–RNA contacts required for mRNA splicing [114]. The function of archaeal Sm/Lsm proteins is still unknown. In noninfected bacterial cells, Hfq controls the expression of various genes by facilitating imperfect base pairing between small regulatory RNAs (sRNAs) and their mRNA targets [115,116,117,118], acting as an RNA chaperone. Presumably, the role of Hfq is not limited to the control of gene expression, as there is evidence that the RNA chaperone activity of Hfq is required in other cellular processes. It was shown that Hfq was involved in mRNA polyadenylation [119], regulation of virulence-factor synthesis [120], and even the formation of the bacterial nucleoid [121].

Recent data revealed a novel role for Hfq. Andrade and coauthors demonstrated that hfq knockout in *E. coli* conferred the cold-sensitive phenotype (slow growth of the Δhfq cells at 16 °C), which is a hallmark of ribosome biogenesis defects. The Hfq-deleted mutant accumulated free 30S subunits and the pre-16S rRNA; the 70S ribosome pool was reduced [122]. In addition, in this mutant strain, translation efficiency and fidelity were impaired: an increased frequency of frame shift, translation initiation on alternative start codons, and premature translation termination were observed.

It was shown that trans expression of the recombinant Hfq suppressed the defects of the 30S subunit maturation. Analysis of Hfq protein mutants revealed that only one of the three RNA-binding sites is critical for ribosome biogenesis: mutations of the distal face of Hfq led to altered ribosomal profiles [106]. These profiles were similar to that of the *hfq* null mutant. It was demonstrated that Hfq interacted with the 5′- and 3′-regions of the 17S rRNA [122]. In addition, Hfq and RNase R can form a complex, and cosediment with 30S subunits [123]. Double inactivation of the Hfq and RNase R genes (Δ*hfq* Δ*rnr* mutant) led to notable cell growth defects and more substantial accumulation of unprocessed rRNAs compared to the Δ*hfq* mutant. These results indicated that RNase R interacts functionally with Hfq in the ribosome biogenesis. RNase R is highly effective against structured RNAs [124]. Cooperation between Hfq and RNase R may help eliminate RNA secondary structures and make the 5′- and 3′-ends available for folding, promoting the correct processing of 17S rRNA. Moreover, it has been reported that Hfq and RNAse R can associate with the ribosomal protein S12, which affects translation fidelity and is located close to the decoding center [125]. These findings indicated that Hfq and RNase R could interact with the pre-30S subunit through S12 and form the productive structure of the 17S rRNA 5′- and 3′-sequences, which are part of the decoding center.

### 2.9. Network of 30S Assembly Factors

**Figure 6:**
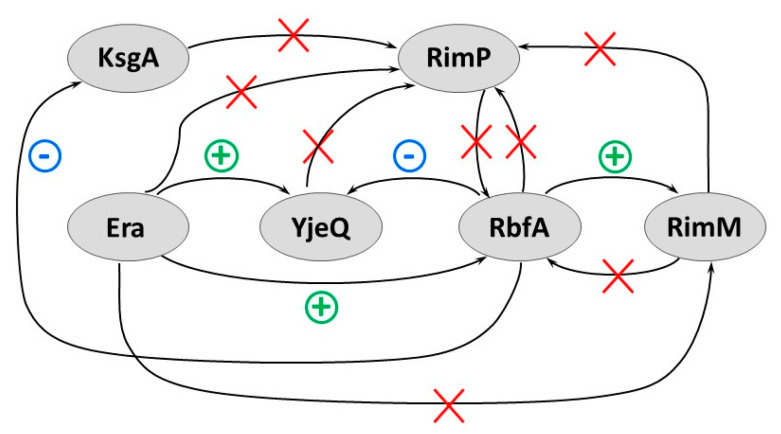
Network of functional interactions between the 30S assembly factors. Effects of the overproduction of one assembly factor on the defects caused by the lack of another one are shown by arrows. A plus sign indicates a positive effect, a minus sign indicates a negative effect, and a cross (×) signifies that no effects were observed. A positive effect implies the partial suppression of the defects of ribosome assembly. A negative effect implies the exacerbation of the defects of ribosome assembly. This network is described in the main text in more detail.

Here, we systematized the biochemical and genetic data on the described above ribosome biogenesis factors to build up a network of functional interactions between the 30S assembly factors (Figure 6). In this scheme, the arrows connecting two factors indicate negative (−), positive (+), or lack (×) of the effect on the ribosome biogenesis in a strain deleted for one factor (located at the arrowhead) by an excess of another factor provided in trans (located at the tail). Positive relationships (synergy or additivity) indicate that functions of the two factors either overlap or are related to each other; whereas in the case of negative relationships, factors act as antagonists. Such relationships imply both functional redundancy and certain hierarchy for some of the ribosome biogenesis factors. This network, along with structural analysis of assembly intermediates, reveal a temporal framework and an order of action of these factors during the maturation of the CDR. Therefore, we described the time course of the events during the CDR maturation assisted by the selected 30S assembly factors. We propose the following sequence of the factor actions: RimM + Era > RbfA > RimP > RimJ > YjeQ > KsgA > Hfq. Factors RimM, Era, RbfA, RimP, and RimJ were assigned to a group of the “early”-stage factors that take part in the formation of the central pseudoknot, including folding of the head. Some “early”-stage factors assist the CDR maturation throughout the entire process; for example, RimP and Era. The remaining factors (YjeQ, KsgA, and Hfq) belong to a group of “late”-stage factors, which are required for positioning of the helix 44 and processing of the 5′- and 3′-ends of the 16S rRNA.

Ribosome biogenesis occurs in the 5′-to-3′ direction. We suggest that the “early”-stage factors of RimM and Era are among the first to take part in the 30S subunit assembly. It appears that RimM binds the 3′ major domain cotranscriptionally, together with protein S19, and facilitates the assembly of the head. At the same time, Era interacts with the 3′-end of the 17S rRNA during the excision of the 16S rRNA precursor from the primary transcript and protects the anti-SD sequence from cleavage. The early association of Era with the 3′-end of the 17S rRNA and the Era-promoted platform stabilization may induce the movement of the 3′-end to the exit of the maturing mRNA channel. In turn, it facilitates the RbfA binding to the pre-30S subunits. As a gatekeeper, RbfA is bound within the mRNA channel exit, where it captures the 3′-end region of the 17S rRNA upstream of the Era-binding site. Therefore, RbfA blocks the mRNA interaction with the pre-30S subunit, preventing premature translation, as does Era.

The positioning of the 3′-end of the 17S rRNA at the exit of the mRNA channel is accompanied by conversion of h28 into the mature form. This conversion is assisted by RbfA. The assembly factor RbfA distorts the upper region and destabilizes the lower region of h28. These conformational changes in the helix 28 impede folding of the h44/45 linker. Apparently, the delay in the folding of the h44/45 linker is required for the formation of central pseudoknot (PK) helices 1 and 2. The accurate formation and stability of the central PK is a critical step during the small subunit assembly. The central PK connects the head, body, and platform with each other. During the early stages of the 30S subunit biogenesis, premature central PK formation is blocked by a structure within the unprocessed 5′-region (the 5′-leader sequence) of the 17S rRNA that is mutually exclusive with h1. During the CDR folding, RbfA is implicated in the restructuring of the 5′-leader sequence of the 17S rRNA. RbfA is thought to refold the 5′-leader sequence via h28 and to promote formation of h1. It may also act synergistically with RimP to stabilize the central PK. RimP plays a key role in the central PK stabilization, accelerating the subsequent incorporation of central PK-binding r-proteins S5 and S12. Obviously, RimJ acetylates S5 at this stage, signaling that the central pseudoknot is formed. RimJ may dissociate after acetylation. We cannot rule out that RimJ is also directly involved in the central PK stabilization—the remodeling the structure of the central pseudoknot for stable association of protein S5 with its subsequent acetylation.

Then, the formed central pseudoknot anchors the head domain to the body and platform. At that point, docking of the head to the platform domain may occur either with an assembled head domain or with one only partially formed. Therefore, the RimM-promoted folding of the 3′ major domain and RbfA-supported conformational changes in the 5′ domain required for the formation of the central pseudoknot may occur additively. However, dissociation of RimM occurs prior to stabilization of the central PK incorporation of S12. RimP appears to remain on the pre-30S subunit until the final stages of the 17S rRNA processing. It binds to S12 so that h44 cannot access its native position, keeping the upper part of h44 and its linker regions disordered. Thus, RimP maintains the mRNA channel unstructured in the neck region for the rearrangement of the 3′-end region of the 17S rRNA. It has been considered that RimP exposes the KsgA binding site and may even preassociate with KsgA to facilitate its recruitment to the subunit.

The checkpoint proteins KsgA and YjeQ are involved in the CDR maturation after the formation of the central PK and docking of the head to the platform domain. YjeQ binds to the head and neck of h28 and the top of helix 44 and probes the state of maturating 30S subunits. YjeQ induces release of RbfA. As a result, the 3′-end region of the 17S rRNA is free to rearrange near the neck and interact with h28 to stabilize the h44/45 linker. YjeQ further promotes h44/45 linker maturation. It clamps around the top of the h44, fixing the positions of A1492, which monitors the geometry of the codon–anticodon base pairing during the translation, and of the adjacent adenosine A1493. Limiting the h44/45 linker-folding landscape favors interactions with the minor groove of h44. After that, the GTP-activity is triggered, and YjeQ dissociates from the pre-30S subunit. Then, quality of the CDR assembly is monitored by the methyltransferase KsgA, keeping h44 from containing the h45 tetraloop. Dimethylation of A1518 and A1519 induces the packing interaction between helices 45 and 44. The methylation is assumed to be a trigger for the release of KsgA from assembling 30S subunits. Most likely, the release of KsgA is accompanied by Era dissociation and RimP exchange for Hfq, which are required for the final stages of the 17S rRNA processing. Hfq, along with RNAses, form the productive structure of the 5′- and 3′-ends of the16S rRNA. The final h44/h45 docking on the front of the 30S subunit occurs after the trimming of the 5′- and 3′-ends of the 17S rRNA and the release of Hfq/RNAses. This allows the 30S subunit’s entrance into the translation cycle.

## 3. Conclusions

Currently, the understanding of ribosome assembly is rather poor as compared to other aspects of ribosome functioning. The delay in the field of ribosome biogenesis was due to a lack of techniques that allowed studying this process dynamically in vivo. Moreover, isolation of ribosome-assembly intermediates from wild-type cells is tedious, as they are short-lived and not abundant. Investigation of the ribosome assembly began with the works of Nomura and colleagues, who built the map of the reconstitution of the 30S subunit in vitro from individual 16S rRNA and ribosomal proteins [126,127,128,129]. These studies demonstrated the complexity of the 30S subunit assembly: incorporation of ribosomal proteins into maturing 30S subunits occurs in a hierarchical and cooperative manner. The development of new biophysical techniques has provided valuable information on the kinetics and mechanism of rRNA folding in subunit assembly in vitro [130,131]. Moreover, combination of these techniques with genetic approaches uncovered several factors that facilitate biogenesis processes and shed light on numerous nuances regarding the order of incorporation of the ribosomal proteins into the forming ribosome [8,100,132,133]. This suggests that in living cells, the ribosome biogenesis does not occur as it was demonstrated in the in vitro experiments. Inactivation of genes of proteins that participate in the ribosome biogenesis by modern genetic methods allows obtaining in vivo ribosomal-assembly intermediates that are “frozen” at the stage that requires participation of the corresponding protein. Analysis of these intermediates showed steps of the ribosome assembly in cells, and the functions of proteins that assisted in the corresponding steps. This, in its turn, provided an understanding of the ribosome maturation mechanism in more detail. Currently, a number of proteins related to ribosome biogenesis have been identified whose functions are unknown. These are yet to be integrated into the complex network of participants of this process.

It is worth mentioning that during the era of multidrug resistance in bacteria, studying the ribosome assembly becomes a more important task than ever. Previously, the inhibition of the translation with antibiotics was studied substantially. At the time, this allowed the development of a wide range of antimicrobial molecules. Ubiquitous application of this tool against pathogenic bacteria resulted in the appearance of bacteria that were resistant to conventional antibiotics. This state of affairs urges researchers to find new chemicals that can kill or stunt bacteria by targeting other fundamental processes. Affecting cellular processes requires understanding the principles of their regulation. Thus, studying the regulation of the ribosome assembly is one of the promising fields. The knowledge of the molecular mechanisms driving ribosome assembly is essential for designing new pharmaceutical approaches to combat multidrug resistant pathogenic bacteria and understanding the mechanistic basis of ribosomopathies in humans.

## Data Availability

All data referenced in this review were reported in PubMed-indexed literature available online.
